# Evaluating the Efficacy of Rose Bengal as a Photosensitizer in Antimicrobial Photodynamic Therapy Against *Candida albicans*: A Systematic Review

**DOI:** 10.3390/ijms26115034

**Published:** 2025-05-23

**Authors:** Jakub Fiegler-Rudol, Barbara Lipka, Katarzyna Kapłon, Magdalena Moś, Dariusz Skaba, Aleksandra Kawczyk-Krupka, Rafał Wiench

**Affiliations:** 1 Department of Periodontal Diseases and Oral Mucosa Diseases, Faculty of Medical Sciences in Zabrze, Medical University of Silesia, 40-055 Katowice, Poland; jakub.fieglerrudol@gmail.com (J.F.-R.); s81112@sum.edu.pl (B.L.); dskaba@sum.edu.pl (D.S.); rwiench@sum.edu.pl (R.W.); 2 Department of Internal Diseases, Angiology and Physical Medicine, Centre for Laser Diagnostics and Therapy, Medical University of Silesia, Batorego 15, 41-902 Bytom, Poland; katarzyna.kaplon.kk@gmail.com (K.K.); magdalenamos99@gmail.com (M.M.)

**Keywords:** aPDT, biofilm, *Candida*, denture stomatitis, diode laser, planktonic cells

## Abstract

*Candida albicans* is a significant pathogen in various fungal infections, including oral candidiasis and denture stomatitis. As antifungal resistance rises globally, there is an urgent need for alternative treatment strategies. Antimicrobial photodynamic therapy (aPDT), utilizing a photosensitizer and light to produce reactive oxygen species (ROS), has emerged as a promising approach. Rose Bengal (RB), a xanthene dye, exhibits a high singlet oxygen quantum yield, making it a candidate for aPDT. However, its efficacy in *C. albicans* treatment has been inconsistent, particularly against biofilm-associated infections, which are more resistant to conventional therapies. This systematic review evaluates the efficacy of Rose Bengal-mediated aPDT in combating *C. albicans* infections by synthesizing data from studies conducted over the past decade. We focus on the effectiveness of RB across different experimental conditions, including planktonic and biofilm forms of *C. albicans*. The review also explores the synergy between RB and other agents, such as potassium iodide, and compares the outcomes of RB-mediated aPDT to other photosensitizers and conventional antifungal treatments. Despite its potential, RB-aPDT shows variable effectiveness due to differences in experimental protocols, such as the photosensitizer concentration, incubation times, and light parameters. The review identifies the key limitations, such as RB’s poor biofilm penetration and high dark toxicity at elevated concentrations, which hinder its clinical applicability. The combination of RB with potassium iodide enhances its antifungal efficacy, suggesting that further optimization could improve its clinical potential. Overall, while Rose Bengal-mediated aPDT holds promise as a novel antifungal treatment, further research is needed to standardize protocols, enhance delivery systems, and validate its efficacy in vivo and clinical settings.

## 1. Introduction

### 1.1. Rationale

Fungal infections pose a significant global health challenge, with *Candida albicans* being among the most prevalent opportunistic pathogens implicated in both superficial and systemic diseases [1,2,3]. Clinically, *C. albicans* stands out as a principal etiological factor in oral and oropharyngeal candidiasis, including denture stomatitis, as well as in life-threatening invasive candidiasis [4,5,6,7]. While conventional antifungals—such as azoles, polyenes, and echinocandins—continue to play a major role in treatment, they exhibit drawbacks that hinder optimal patient outcomes [8,9]. These drawbacks include drug-related toxicity, drug–drug interactions, and, critically, the rising issue of antifungal resistance [10]. Indeed, the World Health Organization has highlighted the urgent need to address antifungal resistance, noting that its global burden has profound implications for morbidity, mortality, and healthcare costs [11]. Against this backdrop, antimicrobial photodynamic therapy (aPDT) has emerged as a promising alternative or adjunctive modality for fungal eradication [12,13]. The central premise of aPDT lies in the combined action of a photosensitizer (PS) and a specific wavelength of light, resulting in the production of cytotoxic reactive oxygen species (ROS) that can damage cellular components—lipids, proteins, and nucleic acids—ultimately leading to microbial cell death [14,15,16,17]. A critical advantage of aPDT is that it targets fundamental microbial structures and pathways that are less prone to mutation-driven resistance [16]. Consequently, repeated aPDT treatments are less likely than conventional antifungals to select for resistant strains, a factor of increasing importance in clinical microbiology [14]. Chemically classified as a xanthene dye, RB has a high singlet oxygen quantum yield, making it particularly effective in generating the ROS necessary for microbial inactivation [17]. Originally employed in ophthalmology and histological staining, RB’s transition into infection control and wound management is fueled by its strong photochemical activity under green to yellow light (approximately 515–570 nm) [17,18]. However, its application in clinical dentistry remains somewhat sporadic. The key factors contributing to the inconsistent outcomes observed in studies of RB-mediated aPDT include variations in photosensitizer concentration, incubation times, and light source parameters (wavelength, energy density, and fluence) [19,20]. These discrepancies complicate the establishment of standardized protocols and hinder direct comparisons across research findings [19,20]. Moreover, many investigations focus predominantly on planktonic cells of *C. albicans*, whereas biofilm-associated infections—common in denture stomatitis, endodontic pathologies, and medical device-related infections—are often more resistant to treatment [21]. Biofilms create physical and biochemical barriers that limit the penetration and efficacy of both antifungal agents and the ROS generated during aPDT [22]. Thus, understanding how RB-mediated aPDT performs in the context of biofilms is crucial for translating in vitro efficacy into real-world clinical success [23]. In addition to biofilm challenges, studies exploring the synergistic or adjuvant use of RB with other agents—such as potassium iodide—have shown that the overall antimicrobial effect can be substantially enhanced [23]. These findings point to the potential for combined or sequential therapeutic strategies that could further improve aPDT outcomes. Given the urgent demand for antifungal therapies that circumvent the pitfalls of current pharmacological options, consolidating the literature on RB-mediated aPDT becomes imperative [15,16,17,18,19,20,21,22,23,24,25,26,27,28,29,30].

### 1.2. Objectives

This systematic review aims to evaluate the efficacy of Rose Bengal as a photosensitizer in aPDT for the treatment of *C. albicans* infections. By rigorously analyzing data from studies conducted over the past decade, we seek to compare the antifungal outcomes of Rose Bengal-mediated therapy with alternative treatments, including blue light irradiation alone and other photosensitizing agents. Ultimately, our review endeavors to clarify whether Rose Bengal-mediated aPDT can serve as a viable alternative or adjunctive strategy to conventional antifungal treatments, thereby informing future research directions and clinical practices in the management of *Candida*-associated infections.

## 2. Materials and Methods

### 2.1. Focused Question

A systematic review was conducted in accordance with the PICO framework [31], adapted for in vitro research models. The focused question was: In in vitro models simulating superficial Candidiasis caused by *C. albicans*, does treatment with Rose Bengal-mediated antimicrobial photodynamic therapy (RB-aPDT), compared to blue light irradiation alone, RB without light activation, or conventional antifungal agents, result in a greater reduction or eradication of *C. albicans*?

### 2.2. Search Strategy

**Figure 1:**
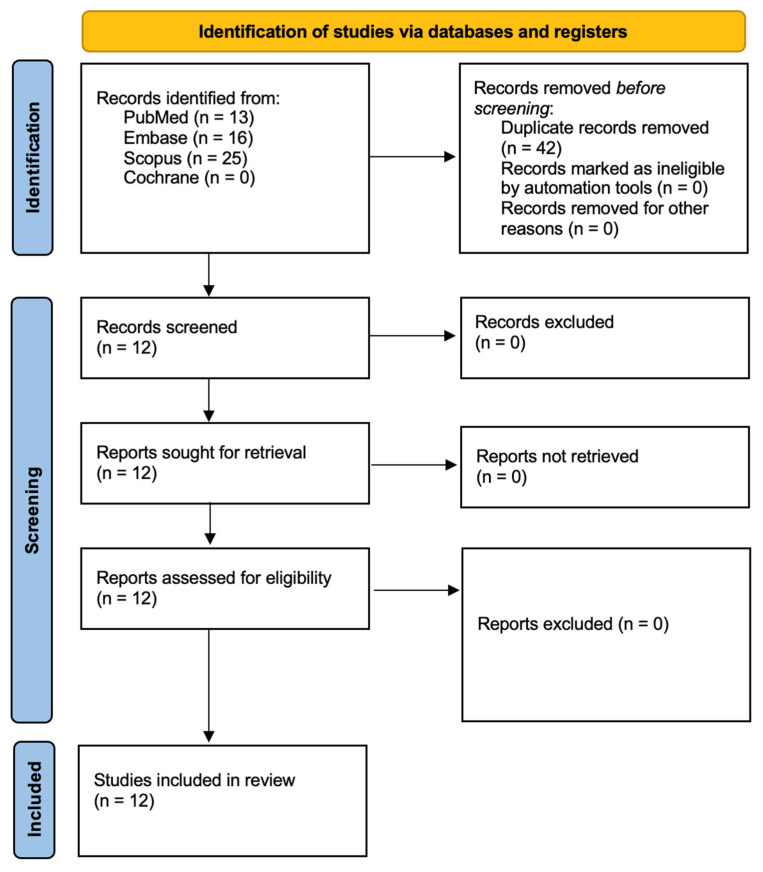
Prisma 2020 flow diagram.

This systematic review has been registered with PROSPERO under the assigned ID CRD420250651918. The study adhered to the Preferred Reporting Items for Systematic Reviews and Meta-Analyses (PRISMA 2020) guidelines [32]. A comprehensive literature search was conducted across multiple electronic databases, including PubMed/Medline, Embase, Scopus, and the Cochrane Library, with detailed search strategies outlined in Figure 1. Three independent researchers performed the database searches using consistent search terms. To refine the selection, additional electronic filters were applied, limiting the results to studies published in English between 1 January 2014, and 3 December 2024. The initial screening was based on titles and abstracts, ensuring an alignment with predefined inclusion criteria (Table 1). Subsequently, two authors conducted a full-text review of the shortlisted studies to extract the relevant data. To further enhance the scope of the review, a snowball search was employed, examining the reference lists of eligible studies to identify additional relevant literature. This review aimed to evaluate the potential of Rose Bengal-mediated antimicrobial photodynamic therapy as an effective strategy for *Candida* eradication, exploring its viability as an alternative or adjunctive treatment to conventional pharmacological approaches. Studies included in the final analysis were selected based on clearly defined inclusion and exclusion criteria.

### 2.3. Selection of Studies

To ensure an unbiased selection process in this systematic review, the reviewers independently assessed the titles and abstracts of all retrieved studies. Any discrepancies regarding study eligibility were resolved through thorough discussions until a consensus was reached. This meticulous approach, in compliance with PRISMA guidelines, was designed to enhance the rigor of the review by incorporating only the most relevant and methodologically sound studies. By applying these stringent selection criteria (Table 2), the reliability and reproducibility of the findings were reinforced, ensuring a comprehensive evaluation of Rose Bengal-mediated antimicrobial photodynamic therapy in *Candida* treatment [32].

### 2.4. Risk of Bias in Individual Studies

In the initial phase of the study selection, the reviewers independently assessed the titles and abstracts of the identified studies to reduce potential bias in the screening process. To evaluate the consistency of their assessments, Cohen’s kappa statistic was employed as a measure of inter-reviewer agreement [33]. Any discrepancies regarding study inclusion or exclusion were resolved through detailed discussions among the authors until a consensus was reached.

### 2.5. Quality Assessment and Risk of Bias Across Studies

The quality of the included studies was independently assessed by three authors, focusing on critical elements of aPDT design, implementation, and data analysis to ensure objectivity and validate findings. The risk of bias was determined by assigning a score of 1 for each “yes” and 0 for each “no” response to the following criteria: (1) Was a specific concentration of Rose Bengal as the photosensitizer indicated? (2) Was the origin or source of Rose Bengal provided? (3) Was the incubation time clearly stated? (4) Were detailed parameters of the light source (e.g., type, wavelength, output power, fluence, power density) provided? (5) Was a power meter used in the study to confirm light dose? (6) Was a negative control group included in the experimental design? (7) Were numerical results reported with appropriate statistics? (8) Was there no missing outcome data? (9) Was the study independent of its funding source? Each study was scored based on its total number of “yes” responses, then categorized according to predefined thresholds (high risk of bias: 0–3; moderate risk of bias: 4–6; low risk of bias: 7–9). Finally, each study was assigned an overall risk of bias (low, moderate, or high) in accordance with the guidelines set forth in the Cochrane Handbook for Systematic Reviews of Interventions [34]. The results of this quality assessment can be found in Table 3.

### 2.6. Data Extraction

Once an agreement was reached on the selected articles for inclusion, both authors systematically extracted the relevant information. This included the citation details (first author and publication year), the study design, the *Candida* strains analysed, the composition of test and control groups, the follow-up duration, the recorded outcomes, the characteristics and parameters of the light source, the concentration of Rose Bengal, and the presence of additional substances, as well as the incubation and irradiation durations.

## 3. Results

### 3.1. Study Selection

Figure 1 illustrates the study selection process, adhering to PRISMA guidelines [32]. The initial database search identified 54 articles, which was reduced to 12 after removing duplicates. A screening of the titles and abstracts resulted in 12 studies being reviewed in full, with none excluded. Consequently, 12 articles published within the last decade were incorporated into the final analysis. A comprehensive summary of these studies is presented in Table 4.

### 3.2. Data Presentation

Table 4, Table 5, Table 6 and Table 7 present a concise compilation of the information retrieved from the 12 studies that fulfilled the inclusion criteria and were incorporated into the review.

### 3.3. Characteristics of Light Sources Used in aPDT

Table 6 outlines the physical parameters of the light sources used in the studies that satisfied the inclusion criteria. Table 7 shows the characteristics of RB in each study.

The incubation times were standardized to minutes, and Rose Bengal concentrations were uniformly converted to µg/mL using the molecular weight of RB (1017.64 g/mol), where 1 µM equals approximately 1.01764 µg/mL, and 1% *w*/*v* corresponds to 10,000 µg/mL [35].

## 4. Discussion

### 4.1. Results in the Context of Other Evidence

Rose Bengal (RB) has been extensively studied for antimicrobial photodynamic therapy (aPDT) against *C. albicans*, demonstrating consistent antifungal activity—particularly against planktonic cells—due to its strong singlet oxygen generation [35,36,39,43]. However, achieving significant colony-forming unit (CFU) reductions often requires relatively high photosensitizer concentrations and tightly controlled irradiation parameters [35,36,39,43]. A notable advancement is the suggested synergy observed when RB is combined with potassium iodide (KI), which significantly enhances fungal cell death and highlights the potential for more potent photodynamic systems targeting *C. albicans* [35,36]. This proposed synergistic effect could help address limitations of RB monotherapy, such as the need for high doses or extended exposure times. Despite promising in vitro results, the RB–KI strategy remains controversial, as the underlying mechanisms—likely involving reactive iodine species and prolonged reactive oxygen species (ROS) generation—are not fully understood and appear highly dependent on variables like pH, oxygen levels, and photosensitizer concentration. Additionally, concerns about cytotoxicity to host tissues, instability of iodine intermediates, and inconsistent efficacy across microbial species and biofilm models have been raised. Consequently, while the RB–KI combination shows therapeutic promise, further mechanistic studies and rigorous in vivo investigations are essential to determine its safety, reproducibility, and clinical utility [35,36].

The antifungal mechanism of RB-PDT primarily involves the generation of reactive oxygen species (ROS), particularly singlet oxygen (^1^O_2_), which induces oxidative stress and damage to key cellular components such as the fungal cell membrane, mitochondria, and nucleic acids, ultimately leading to cell death [13,14,15]. ROS disrupt membrane integrity by peroxidizing lipids, impair mitochondrial function by altering membrane potential, and fragment DNA, collectively compromising cell viability and proliferation [13,14,15]. Singlet oxygen and other ROS, such as superoxide anions and hydroxyl radicals, induce oxidative stress that compromises fungal cell viability. These species initiate lipid peroxidation, leading to the disruption of membrane integrity and increased permeability, while also damaging the mitochondrial membranes, impairing the energy production, and triggering apoptotic pathways. Additionally, ROS oxidize fungal proteins and fragment DNA, resulting in loss of cellular function and eventual cell death [35,36,37,38,39,40,41,42,43].

Despite these benefits, some studies report that RB may underperform relative to methylene blue (MB) in certain experimental conditions or require higher doses to reach similar fungicidal outcomes [35,36,42]. The variability in light sources—whether LEDs or filtered lamps—and the different wavelengths (ranging from approximately 515 to 660 nm) further complicate the direct comparison of the results across studies [36,37,41,42]. Additionally, essential parameters like the fluence (J/cm^2^), the power density (mW/cm^2^), and the incubation time often differ, which can substantially impact efficacy and limit the establishment of standardized guidelines. Optimizing these parameters is critical to making RB-mediated aPDT a more robust, repeatable, and clinically viable therapy.

Biofilms pose a major disadvantage for RB-based therapies, with several studies reporting that while RB significantly reduces planktonic *C. albicans*, it demonstrates limited penetration and only modest CFU reductions within biofilm matrices [39,41]. This finding underscores the intrinsic difficulty in eradicating biofilm-embedded pathogens. Novel strategies, such as polymeric immobilization or the use of RB diacetate, show promise for enhancing the delivery and retention of the photosensitizer, enabling more effective disruption of biofilms [40,45]. However, such approaches may also raise manufacturing costs or add complexity in terms of storage and handling, illustrating the trade-offs between improved efficacy and practical feasibility.

Repetitive or fractional irradiation protocols emerge as effective solutions to mitigate potential cytotoxicity and cost-related disadvantages. By lowering the overall RB concentration and reducing the total light fluence while applying multiple short exposures, researchers have demonstrated that robust antifungal outcomes can still be maintained [36]. This approach may pave the way for more patient-friendly treatment schemes that minimize adverse effects while preserving efficacy. Some fungal species, such as *Aspergillus fumigatus* and *Curvularia lunata*, remained resistant under particular protocols, hinting that further pathogen-specific refinements are necessary to optimize RB-aPDT for diverse clinical scenarios [44]. Nonetheless, RB-based aPDT has proved especially promising against drug-resistant *Candida* isolates, achieving substantial reductions in CFU counts, a critical advantage given the global rise in antifungal resistance [43].

Beyond the RB-focused research, other studies underscore aPDT’s broad utility. Leila Gholami et al. demonstrated that aPDT effectively lowers pathogenic bacterial loads in periodontal and peri-implant contexts [47,48,49,50,51], while Du et al. showed that combining MB with KI helps reduce antifungal resistance in AIDS patients suffering from oral candidiasis [52]. Gonzales et al. confirmed that aPDT could shorten treatment times and curb healthcare costs for biofilm-associated Candidiasis [53]. Similarly, work by Dias et al. revealed that repeated aPDT sessions using Photodithazine (PDZ) successfully eradicated *C. albicans* in both planktonic and biofilm forms, highlighting the importance of repeated treatment as a strategy for comprehensive elimination of the pathogen. Uddin et al. found that encapsulated Rose Bengal (RB) nanoparticles significantly enhance the efficacy of photodynamic therapy (PDT) in treating triple-negative breast cancer (TNBC) cells, applying such modifications in dental settings may give promising results [54,55,56,57]. Given the heterogeneity among *Candida* species, future studies should prioritize strain-specific optimization to ensure reproducible and clinically meaningful RB-aPDT results [57,58,59,60,61,62,63]. Comparisons between Rose Bengal and other photosensitizers, such as hypericin or new-generation porphyrins, could elucidate which agents offer the best safety-efficacy profile and should be a direction of research.

While RB-aPDT demonstrates clear advantages—including strong photosensitizing properties and efficacy against resistant strains—its practical implementation can be limited by the necessity for higher photosensitizer concentrations, precise light parameters, and the persistent challenge of biofilms. Establishing standardized protocols, particularly with respect to adjuvant compounds like KI and novel delivery strategies, stands out as a vital next step. Indeed, the majority of current studies call for more clinical trials and further in vivo research to substantiate RB’s therapeutic potential and refine usage parameters, ultimately aiming to integrate RB-aPDT more effectively into mainstream antifungal treatment regimens [28,29,30,31,32,33,34,35,36,37,38,39,60,61,62,63,64,65,66,67].

One critical limitation in the clinical translation of Rose Bengal is its tendency to aggregate at higher concentrations, leading to self-quenching and a marked reduction in singlet oxygen generation efficiency. This phenomenon creates a narrow therapeutic window, wherein RB must be carefully dosed to maintain photodynamic efficacy without compromising its reactive oxygen species output [35,36]. Furthermore, RB exhibits relatively high dark toxicity, which poses additional challenges for safe application—particularly in tissues with limited light accessibility. These factors underscore the importance of ongoing efforts to refine RB formulations, such as encapsulation in nanocarriers or conjugation with hydrophilic moieties, to minimize aggregation and improve the safety profile without diminishing antifungal activity [54,55,56,57].

### 4.2. Limitations of the Evidence

One major limitation affecting the strength of the evidence lies in the significant heterogeneity of the included studies, which used varied photosensitizer concentrations, wavelengths, irradiation times, and energy densities. These inconsistencies make it difficult to draw clear comparisons and impede the establishment of uniform protocols for Rose Bengal-mediated antimicrobial photodynamic therapy (aPDT). Additionally, while most investigations demonstrated promising outcomes against *C. albicans*, the bulk of the data remain confined to in vitro settings, with relatively few in vivo or clinical trials to confirm effectiveness under actual physiological conditions. The studies also relied primarily on colony-forming unit (CFU) reduction or qualitative biofilm assessments, often omitting advanced imaging or molecular techniques that could provide a deeper insight into the mechanisms of fungal cell damage and biofilm disruption. Finally, although several studies addressed the potential advantages such as cost reduction and synergy with adjuvants like potassium iodide, few provided extensive safety or adverse-effect profiles, underscoring the need for more rigorous, standardized research designs to verify both the efficacy and clinical viability of RB-aPDT.

### 4.3. Limitations of the Review Process

The primary limitation of this review lies in the marked heterogeneity of the included studies, which varied in their methodologies, intervention protocols, and outcome measures. This variability precluded a quantitative meta-analysis and necessitated a narrative synthesis of the data, potentially limiting the comparability of individual findings. Additionally, the exclusion of non-English language studies and grey literature could have omitted relevant data and introduced publication bias. The lack of standardized protocols among different studies—particularly with respect to photosensitizer concentration, irradiation parameters, and biofilm assessment—further complicated consistent appraisal of outcomes. Finally, while the review aimed to apply rigorous selection criteria, potential bias may still stem from the inevitable subjectivity involved in interpreting methodologies and results, underscoring the need for more comprehensive, multicenter clinical trials to reinforce and refine the evidence base.

### 4.4. Implications for Practice, Policy, and Future Research

The findings of this review highlight key implications for clinical practice and future research. First, while Rose Bengal-mediated aPDT has demonstrated consistent antifungal efficacy against *C. albicans*, its applicability across other *Candida* species remains uncertain. Given the genetic, structural, and metabolic variability among *Candida* strains, such as *C. glabrata, C. tropicalis*, and *C. krusei*—future studies must investigate the strain-specific responses to RB-aPDT to avoid overgeneralization and ensure therapeutic relevance. From a policy standpoint, establishing standardized protocols for photosensitizer concentration, irradiation parameters, and treatment regimens is essential for translating promising in vitro results into clinically viable interventions. Furthermore, regulatory bodies and funding institutions should prioritize support for in vivo and clinical trials, especially those comparing RB-aPDT to standard antifungal therapies in localized candidiasis, such as denture stomatitis or oropharyngeal infections. Exploring advanced delivery systems, such as nanocarriers or surface immobilization techniques, may also enhance photosensitizer stability and biofilm penetration, ultimately improving clinical outcomes. Collectively, these directions will be pivotal in validating RB-aPDT as a cost-effective and resistance-mitigating adjunct to conventional antifungal strategies. This review uniquely consolidates the current body of evidence specifically on Rose Bengal-mediated antimicrobial photodynamic therapy (RB-aPDT) against *C. albicans*, the first systematic review to do so with exclusive focus on this photosensitizer-pathogen pairing. While previous analyses have broadly assessed aPDT or compared multiple photosensitizers, none have critically appraised the experimental conditions, fungal targets (planktonic vs. biofilm), nor synergistic strategies (e.g., with potassium iodide) of RB-based protocols in such detail. Despite being rooted in preclinical studies, the findings have direct clinical relevance, especially given RB-aPDT’s demonstrated efficacy against drug-resistant *C. albicans* strains [43,44], its affordability using LED-based systems [36], and its potential as a non-invasive adjunct in treating superficial infections like oral candidiasis or fungal keratitis [39,44]. Nevertheless, several research gaps must be addressed before clinical integration. First, RB-aPDT shows limited efficacy against biofilm-associated *C. albicans*, which poses a significant challenge in denture stomatitis and device-related infections [39,41]. Second, wide methodological variability—spanning RB concentrations, light parameters, and incubation protocols—prevents standardization and reproducibility [36,37,42]. Third, safety concerns persist, particularly RB’s dark toxicity and aggregation at higher doses, emphasizing the need for robust cytotoxicity and pharmacokinetic studies [35,36,54]. Fourth, the current evidence base is dominated by in vitro models, with few in vivo studies available to validate clinical efficacy [43,44]. Finally, advanced delivery systems, such as nanoparticle encapsulation or polymeric matrices, are needed to improve photosensitizer stability, biofilm penetration, and therapeutic precision [40,45,55]. By addressing these limitations through translational and clinical research, RB-aPDT may ultimately emerge as a safe, effective, and resistance-mitigating strategy in the antifungal treatment landscape.

### 4.5. Research Gaps and Future Research Directions

Several research gaps must be addressed to facilitate the clinical translation of Rose Bengal-mediated antimicrobial photodynamic therapy (RB-aPDT). First, despite its efficacy against planktonic *C. albicans*, RB-aPDT shows limited effectiveness against biofilms, which provide structural and biochemical barriers that impair photosensitizer penetration and reactive oxygen species activity [39,41]. Second, the lack of standardized protocols—especially regarding RB concentration, light wavelength, fluence, incubation times, and irradiation duration—hampers reproducibility and meaningful comparisons across studies [36,37,42]. Third, safety concerns such as RB’s dark toxicity, its tendency to aggregate at high concentrations, and potential cytotoxicity to host tissues necessitate rigorous toxicological evaluation to determine safe and effective therapeutic windows [35,36,54]. Fourth, most evidence is based on in vitro models, with few in vivo or clinical studies available to confirm efficacy under physiological conditions or in disease-specific contexts such as denture stomatitis or keratitis [43,44]. Finally, there is a pressing need to develop optimized delivery platforms—such as nanoparticle carriers or polymeric matrices—to enhance RB bioavailability and clinical usability in antifungal therapy [40,45,55]. Addressing these gaps will be essential for standardizing RB-aPDT protocols, improving safety, and validating its role as a viable adjunct or alternative to conventional antifungal treatments.

## 5. Conclusions

This systematic review consolidates current evidence supporting the antifungal potential of Rose Bengal-mediated antimicrobial photodynamic therapy (RB-aPDT) against *C. albicans*, particularly in its planktonic form. While several studies demonstrated encouraging results—including notable reductions in colony-forming units, a synergy with potassium iodide, and efficacy against drug-resistant strains—the therapeutic outcomes remain highly dependent on experimental parameters such as the photo-sensitizer concentration, the irradiation conditions, and the pre-irradiation incubation times. Crucially, the evidence base is almost exclusively centered on *C. albicans*, with minimal exploration of other clinically relevant *Candida* species such as *C. glabrata*, *C. tropicalis*, and *C. krusei*, each of which possesses distinct virulence factors and antifungal resistance profiles. Given this taxonomic and physiological diversity, future investigations must prioritize strain-specific assessments to determine the broader applicability of RB-aPDT across the *Candida* genus. Standardizing treatment protocols and expanding in vivo and clinical research will be essential to translate promising in vitro results into practical, reproducible therapies. As antifungal resistance continues to escalate globally, RB-aPDT may emerge as a valuable adjunct or alternative, provided that its efficacy is validated across diverse *Candida* strains and optimized for biofilm-associated infections.

## Figures and Tables

**Table 1:** Search syntax used in study.

Source	Search Term	Filters	Number of Results
PubMed	(“Rose Bengal” [Title/Abstract] OR “Rose Bengal-mediated photodynamic therapy” [Title/Abstract] OR “Rose Bengal aPDT” [Title/Abstract]) AND (“Candida albicans” [Title/Abstract] OR “*C. albicans*” [Title/Abstract]) AND (“antimicrobial photodynamic therapy” [Title/Abstract] OR “aPDT” [Title/Abstract] OR “photodynamic inactivation” [Title/Abstract] OR “PACT” [Title/Abstract] OR “Photodynamic Antimicrobial Chemotherapy” [Title/Abstract])	English language Publication years: 2014–2024 Full text	13
Embase	(‘rose bengal’:ti,ab,kw OR ‘rose bengal-mediated photodynamic therapy’:ti,ab,kw OR ‘rose bengal apdt’:ti,ab,kw) AND (‘candida albicans’:ti,ab,kw OR ‘*C. albicans*’:ti,ab,kw) AND (‘antimicrobial photodynamic therapy’:ti,ab,kw OR ‘apdt’:ti,ab,kw OR ‘photodynamic inactivation’:ti,ab,kw OR ‘PACT’:ti,ab,kw OR ‘photodynamic antimicrobial chemotherapy’:ti,ab,kw)	Publication years: 2014–2024 Controlled Clinical Trial Randomized Controlled Trial	16
Scopus	TITLE-ABS-KEY (“Rose Bengal” OR “Rose Bengal-mediated photodynamic therapy” OR “Rose Bengal aPDT”) AND TITLE-ABS-KEY (“Candida albicans” OR “*C. albicans*”) AND TITLE-ABS-KEY (“antimicrobial photodynamic therapy” OR “aPDT” OR “photodynamic inactivation” OR “PACT” OR “Photodynamic Antimicrobial Chemotherapy”)	Article Publication years: 2014–2024	25
Cochrane	(“Rose Bengal” OR “Rose Bengal-mediated photodynamic therapy” OR “Rose Bengal aPDT”) AND (“Candida albicans” OR “*C. albicans*”) AND (“antimicrobial photodynamic therapy” OR “aPDT” OR “photodynamic inactivation” OR “PACT” OR “Photodynamic Antimicrobial Chemotherapy”)	Publication years: 2014–2024	0

**Table 2:** Selection criteria for papers included in the systematic review.

Inclusion Criteria	Exclusion Criteria
Studies investigating *Candida* elimination using Rose Bengal-mediated aPDT in both in vitro and animal models. In vitro and animal studies that examine *Candida* species and their susceptibility to Rose Bengal-based aPDT. Studies where Rose Bengal is used as the primary photosensitizer in aPDT for *Candida* treatment. Studies assessing synergistic effects of Rose Bengal-mediated aPDT in combination with other antifungal agents. Controlled studies evaluating the effects of Rose Bengal-aPDT compared to untreated controls or alternative therapeutic approaches. Comparative analyses examining the efficacy of Rose Bengal-mediated aPDT versus conventional antifungal treatments. Longitudinal studies or those with follow-up periods to assess the sustained antifungal effects of Rose Bengal-aPDT.	Grey literature sources, case reports, letters to editors, narrative or systematic reviews, books, documents, and other non-journal materials. Non-peer-reviewed sources. Studies published in languages other than English. Duplicate studies or research sharing the same ethical approval number. Studies without a control or comparison group. Research on aPDT where it is not applied as a therapeutic intervention for *Candida*. Studies using photosensitizers other than Rose Bengal. Studies focusing on infections other than *Candida* or those that do not specifically assess *Candida* strains. In vitro studies that do not replicate oral conditions relevant to *Candida* infections.

aPDT: Antimicrobial Photodynamic Therapy.

**Table 3:** The results of the quality assessment and the risk of bias across the studies.

Study	1	2	3	4	5	6	7	8	9	Total Score	Bias Risk
Soria-Lozano et al., 2015 [35]	1	0	1	1	0	1	1	1	0	7	Low
Torres-Hurtado et al., 2019 [36]	1	0	1	1	0	1	1	1	0	7	Low
Alhenaki et al., 2021 [37]	1	1	1	1	0	1	1	1	0	8	Low
Diogo et al., 2017 [38]	1	0	1	1	0	1	1	1	0	7	Low
Freire et al., 2013 [39]	1	0	1	1	0	1	1	1	0	7	Low
Gavara et al., 2021 [40]	1	0	1	1	0	1	1	1	0	7	Low
Silva et al., 2016 [41]	1	1	1	1	0	1	1	1	0	7	Low
Tunçcan et al., 2018 [42]	1	0	1	1	0	1	1	1	0	6	Moderate
Hung et al., 2022 [43]	1	0	1	1	1	1	1	1	0	7	Low
Arboleda et al., 2024 [44]	1	1	1	1	1	1	1	1	0	8	Low
Wei et al., 2024 [45]	1	1	1	1	1	1	1	1	0	8	Low
Wen et al., 2017 [46]	1	1	1	1	1	1	1	1	0	8	Low

**Table 4:** *C. albicans* Strains evaluated in each study.

Author and Year	*C. albicans* Strain Evaluated
Soria-Lozano et al., 2015 [35]	ATCC 10231
Alhenaki et al., 2021 [37]	ATCC Strain, number not specified
Freire et al., 2013 [39]	ATCC 18804
Gavara et al., 2021 [40]	ATCC 10231
Silva et al., 2016 [41]	ATCC 18804
Tunçcan et al., 2018 [42]	ATCC 90028
Hung et al., 2022 [43]	ATCC 10231
Wei et al., 2024 [45]	ATCC MYA-2876
Wen et al., 2017 [46]	CEC 749, Luciferase-expressing strain
Diogo et al., 2017 [38]	Pathogenic Yeast Collection of the Faculty of Medicine, University of Coimbra (FMUC), Portugal. No. YP0037
Arboleda et al., 2024 [44]	Clinical isolate recovered from the cornea of a patient with culture-positive fungal keratitis. Ocular Microbiology Laboratory, Bascom Palmer Eye Institute, University of Miami.
Torres-Hurtado et al., 2019 [36]	Clinical isolate donated by the Department of Mycology from Benemérita Universidad Autónoma de Puebla, Mexico

**Table 5:** Main outcomes and details from each study.

Author and Year	Study Groups	Outcomes
Soria-Lozano et al., 2015 [35]	Three experimental groups based on different microorganisms: *Streptococcus mutans* (ATCC 35668), *Streptococcus sanguis* (ATCC 10556), and *C. albicans* (ATCC 10231). Each of these groups was subjected to aPDT using one of three photosensitizers: methylene blue, RB, or CUR in combination with white light. The study evaluated the photodynamic efficacy of each photosensitizer across these three microbial strains at varying concentrations and incubation times.	RB reduced *C. albicans* viability by 5 log_10_, but only at relatively high concentrations (≥320–640 μg/mL), and even higher concentrations were needed when longer pre-irradiation incubation times were applied. Compared to methylene blue, which was more effective at lower concentrations, RB demonstrated limited antifungal efficacy. The study concluded that MB was the most effective photosensitizer for *C. albicans*, while RB showed greater efficacy against *Streptococcus* spp., indicating that the antimicrobial efficacy of each photosensitizer is organism dependent.
Torres-Hurtado et al., 2019 [36]	Four experimental groups based on combinations of microorganism species and photosensitizers. *C. albicans* was treated with MB and RB, while *Trichophyton mentagrophytes* was treated only with MB. For each organism–photosensitizer pairing, the study compared single-dose versus repetitive-dose photodynamic inactivation protocols using varying concentrations of photosensitizers and light energy densities. Control groups included untreated cells, light-only exposure, and dark-incubated cells with photosensitizer but no light.	RB is highly effective as a photosensitizer in PDI of *C. albicans*, outperforming MB under comparable conditions. Specifically, complete inhibition of yeast growth (>99%) was achieved using RB at concentrations as low as 1 μM with 30 J/cm^2^ and 5 μM with 10 J/cm^2^ under single-dose light exposure. Moreover, by applying repetitive low-dose light exposures (e.g., 2–3 irradiations with 30-min dark incubation times), the authors were able to significantly reduce both RB concentration and light energy density while maintaining strong antifungal effects. For example, >90% inhibition was achieved using 1 μM RB and 10 J/cm^2^ with two exposures, which used fewer photons and one-fifth of the RB concentration compared to single-dose setups. The repetitive irradiation strategy appeared to enhance efficacy by allowing oxygen replenishment and cumulative photodynamic damage, without increasing toxicity, and could be advantageous for minimizing side effects in sensitive patients.
Alhenaki et al., 2021 [37]	Four groups of acrylic denture resin specimens contaminated with *Streptococcus mutans*, *Staphylococcus aureus*, *Escherichia coli*, and *C. albicans*. Group 1 was treated with Rose Bengal, Group 2 with Methylene Blue, Group 3 with a Porphyrin Derivative, and Group 4 served as the control group treated with 0.12% CHX. Each group underwent aPDT or chemical disinfection, and the effectiveness of microbial reduction was assessed by counting colony-forming units (CFU/mL).	**RB** was tested at a concentration of **5 µM** activated by **480 nm LED light at 200 mW and 526 mW/cm^2^ for 180 s** against *Candida albicans* ATCC strain on acrylic denture resin. The results showed that **RB was the least effective among the photosensitizers tested**, producing a **mean CFU/mL (log_10_) of 6.15 ± 0.31**, indicating limited antifungal activity. In comparison, the **porphyrin derivative (PD)** achieved **3.67 ± 0.18 log_10_ CFU/mL**, and **0.12% chlorhexidine (CHX)**, used as a positive control, yielded **2.09 ± 0.85 log_10_ CFU/mL**. The authors concluded that RB exhibited **selective antimicrobial efficacy**, being more effective against gram-positive bacteria like *S. aureus* and *S. mutans*, but **not effective for inactivating** *C. albicans* under the tested conditions
Diogo et al., 2017 [38]	The study groups included (1) monospecies biofilms of *E. faecalis*, (2) monospecies biofilms of *C. albicans*, and (3) mixed biofilms of both microorganisms. Each biofilm type was treated using different photosensitizers—toluidine blue O(TBO), rose bengal (RB), TMPyP, and Zn(II)chlorin e6 methyl ester (Zn(II)e6Me)—activated by appropriate LED light sources. These groups were compared to controls and to classical endodontic irrigants: 3% sodium hypochlorite (NaOCl), 2% chlorhexidine (CHX), and 17% EDTA. The treatments were assessed at two irradiation durations (60 and 90 s), with pre-incubation in darkness to ensure cellular uptake of the photosensitizers.	**RB** was tested at **0.1 mg/mL** for aPDT against *C. albicans* monospecies and mixed biofilms, using **green LED light at 557 nm** for **60 or 90 s**. While RB exhibited some capacity to reduce *C. albicans* biofilm biomass, its efficacy was **significantly lower than Zn(II)chlorin e6 methyl ester (Zn(II)e6Me)**, especially after 90 s of irradiation, where Zn(II)e6Me showed **superior biofilm removal (*p* = 0.0079)**. RB performed similarly to toluidine blue O (TBO) and was **less effective than TMPyP** and conventional irrigants like CHX and EDTA. Additionally, RB alone (without light) caused minimal disturbance to *C. albicans* biofilms (10% biomass reduction), confirming the need for light activation. The authors concluded that **RB showed limited efficacy in aPDT against** *C. albicans* **biofilms under the tested conditions**, underscoring the importance of optimizing photosensitizer selection for effective endodontic disinfection.
Freire et al., 2013 [39]	For planktonic cultures, six experimental groups were formed: control (PBS, P−L−), light-only (PBS+LED, P−L+), RB without light (P+L−), RB with light (P+L+), EY without light (P+L−), and EY with light (P+L+), using photosensitizer concentrations from 0.78 to 400 μM. For biofilms, six corresponding groups were tested using 200 μM of each photosensitizer. These groups allowed for comparison of the individual and combined effects of photosensitizer and light exposure on fungal viability.	**RB** was evaluated as a photosensitizer for photodynamic inactivation of *C. albicans* ATCC 18804 using a **green LED (532 ± 10 nm, 16.2 J, 237 mW/cm^2^)**. RB showed significant antifungal activity against **planktonic cells at concentrations of 6.25 μM or higher**, achieving **complete microbial reduction (100%) at 12.5, 25, and 50 μM**. However, in **biofilms**, RB **at 200 μM combined with LED** produced only a **0.22 log_10_ CFU/mL reduction**, significantly less than observed for planktonic forms. Statistical analysis confirmed a significant difference between RB + LED and control (*p* = 0.0057), though the effect was modest. Scanning electron microscopy also showed a visible reduction in fungal structures after RB-mediated PDI. The authors concluded that **RB-PDI is effective against** *C. albicans* **planktonic cells, but less so against biofilms**, likely due to structural and physiological resistance factors inherent to biofilm growth.
Gavara et al., 2021 [40]	The study evaluated the aPDI potential of two rose bengal-loaded cationic polystyrene resins—RB@Pmp (macroporous Amberlite IRA-900) and RB@Pgel (gel-type Amberlite IRA-400)—against five microorganisms: *Escherichia coli*, *Pseudomonas aeruginosa*, *Staphylococcus aureus*, *Enterococcus faecalis*, and *C. albicans*. For each microorganism, ten experimental groups were formed: five under light exposure (RB@Pmp, RB@Pgel, corresponding unloaded resins Pmp and Pgel, and no resin) and five corresponding dark controls. These groups allowed evaluation of both photoactivated and dark antimicrobial effects of the polymers.	**RB** was immobilized on two types of commercial cationic polystyrene resins—Amberlite^®^ IRA-900 (RB@Pmp) and Amberlite^®^ IRA-400 (RB@Pgel)—to create photoactive polymeric materials for antimicrobial photodynamic inactivation (aPDI). Against *C. albicans* ATCC 10231, the materials exhibited **modest antifungal activity**, achieving **reductions of 1.5 to 3.0 log_10_ CFU/mL** after irradiation with green light (515 nm, up to 200 J/cm^2^). However, most of the observed effect (~2.5 log_10_ CFU/mL) was attributed to **dark toxicity of the polymeric matrices**, likely due to the presence of quaternary ammonium groups, with **RB’s direct photodynamic action being minimal**. This aligns with previous findings reporting **scarce photoactivity of RB against** *C. albicans*, possibly due to the yeast’s thick cell wall. The authors concluded that while the system is effective against bacteria, its antifungal efficacy is limited under the tested conditions, but could potentially be improved by increasing RB concentration or modifying the polymer matrix.
Silva et al., 2016 [41]	The P+L+ group was treated with RB at a concentration of 12.5 μM and exposed to LED light at 532 nm with an energy density of 16.2 J; the P+L− group received the same concentration of RB but was not exposed to LED light; the P−L+ group was treated with a 0.9% NaCl solution and exposed to LED light; and the P−L− group, serving as the control, was treated with 0.9% NaCl solution without LED exposure.	**RB at 12.5 μM combined with green LED light at 532 nm and 16.2 J** was tested for its effect on **heterotypic biofilms of** *C. albicans* **(ATCC 18804) and** *Bacillus atrophaeus*. The **photodynamic therapy (PDT) group (P+L+) showed a 33.92% reduction in CFU/mL for** *C. albicans* compared to the control group (P−L−). However, **statistical analysis revealed no significant differences between the PDT group and the control or individual light or photosensitizer-only groups**, indicating that the tested parameters were **insufficient to achieve a meaningful antifungal effect**. The authors concluded that this limited outcome may result from **factors such as the thickness of the** *Candida biofilm*, **photosensitizer concentration, limited light penetration, or inadequate pre-irradiation time**, and emphasized the need for **further optimization of aPDT protocols** targeting *C. albicans* biofilms.
Tunçcan et al., 2018 [42]	The study investigated the in vitro effectiveness of aPDT on biofilms formed by *Staphylococcus aureus*, *Staphylococcus epidermidis*, *C. albicans*, and *Candida parapsilosis*. Study groups were formed by combining three photosensitizers—methylene blue (MB), rose bengal (RB), and riboflavin (RBF)—with corresponding light sources: red LED (660 nm) for MB, green LED (518 nm) for RB, and UVA (370 nm) for RBF. Each microorganism was treated with one of these dye-light combinations, and outcomes were compared to untreated biofilm controls and drug-treated negative controls (amphotericin B or teicoplanin). The biofilm inhibition effects of each combination were evaluated using crystal violet staining, CFU counts, and scanning electron microscopy.	**RB** was evaluated as a photosensitizer aPDT against *C. albicans* (ATCC 90028) biofilms using **green LED light at 518 nm and 0.1% RB concentration**. The combination of green LED and RB produced a **22.7% biofilm inhibition index** in *C. albicans*, indicating **moderate antifungal activity** compared to the **most effective combination—red LED + methylene blue**, which achieved **45.4% inhibition**. The efficacy of green LED + RB was **strain-dependent**, showing no effect against *C. parapsilosis* and variable results against bacterial biofilms. Thus, while RB exhibited some inhibitory effect on *C. albicans* biofilms under specific conditions, it was **less potent than methylene blue** in this APDT setting, suggesting limited standalone utility and the need for optimization in future applications.
Hung et al., 2022 [43]	The study investigated the effectiveness of rose bengal-mediated antimicrobial photodynamic therapy (RB-aPDT) against multidrug-resistant *C. albicans*. The study included four experimental groups: (1) absolute control (no rose bengal, no light), (2) dark control (rose bengal without light exposure), (3) light control (light exposure without rose bengal), and (4) RB-aPDT (rose bengal with light exposure). These groups were subjected to varying incubation times and light fluences (e.g., 10, 20, and 30 J/cm^2^), and the fungal viability was assessed through colony-forming unit (CFU) counts after treatment.	**RB-aPDT** effectively inhibited **multidrug-resistant** *C. albicans* **(BCRC 21538/ATCC 10231)** in a **dose-dependent manner** using a **0.2% RB solution** combined with **green LED light at 540 nm**. The optimal condition—**30 J/cm^2^ light fluence**—resulted in a **4-log_10_ (99.99%) reduction in fungal colony-forming units (CFU)**. Control groups (RB alone, light alone, or no treatment) showed negligible effects, confirming that fungal inhibition required both RB and light. Additionally, RB uptake by *C. albicans* was time-dependent, with strong red fluorescence observed inside the cells after 15–30 min of incubation. These findings indicate that RB-aPDT is a **promising alternative treatment** for drug-resistant *C. albicans*, especially due to its efficacy and the simplicity and affordability of the LED-based system used.
Arboleda et al., 2024 [44]	RB-aPDT on seven fungal keratitis isolates—*Aspergillus fumigatus*, *C. albicans*, *Curvularia lunata*, *Fusarium keratoplasticum*, *Fusarium solani*, *Paecilomyces variotii*, and *Pseudallescheria boydii*. The study groups were defined by combinations of rose bengal concentrations (0.1%, 0.05%, and 0.01%) and irradiation energy levels (5.4 J/cm^2^, 2.7 J/cm^2^ continuous, 2.7 J/cm^2^ pulsed, and 1.8 J/cm^2^), along with a non-irradiated control group for each organism. Each fungal species was tested in triplicate under these varying conditions to assess antifungal efficacy.	The researchers tested three concentrations of RB (0.1%, 0.05%, 0.01%) combined with varying green light irradiation energies (5.4, 2.7, and 1.8 J/cm^2^), using both continuous and pulsed light modes. *C. albicans* was identified as the most susceptible species, exhibiting complete growth inhibition across all ten treatment combinations, including the lowest tested RB concentration (0.01%) and minimal energy dose (1.8 J/cm^2^). This confirmed that *C. albicans* could be effectively inactivated with reduced PDAT parameters, suggesting a high sensitivity to RB-mediated photoinactivation. Notably, RB alone or light alone did not produce antifungal effects, reinforcing the requirement of both components for efficacy. These findings highlight the potential of RB-PDAT as a tailored, low-toxicity approach for treating *C. albicans* keratitis.
Wei et al., 2024 [45]	The study involved both in vitro and in vivo experimental groups to evaluate the antimicrobial and wound-healing effects of rose bengal diacetate (RBDA)-mediated photodynamic inactivation (aPDI), with and without potassium iodide (KI). In vitro, the study used microbial suspensions of *MRSA*, *Escherichia coli*, and *C. albicans*, divided into six groups: (1) blank control (no RBDA, no light), (2) RBDA only (no light), (3) KI only (no light), (4) RBDA + KI (no light), (5) RBDA + light, and (6) RBDA + KI + light. In vivo, diabetic mice with MRSA-infected wounds were assigned to four groups: (1) PBS control, (2) RBDA only, (3) RBDA + light, and (4) RBDA + KI + light. These groups allowed the authors to assess the individual and combined effects of RBDA, KI, and light exposure on microbial killing and wound healing.	*C. albicans* SC5314 (ATCC MYA-2876) was treated with Rose Bengal diacetate for aPDI using green light at 540 nm (10 J/cm^2^). The results showed that RBDA alone, even at 15 µM, exhibited no significant antifungal activity. However, when 100 mM potassium iodide was added, 10 µM RBDA killed 3 log_10_ units and 15 µM RBDA killed over 4 log_10_ units of *C. albicans*, demonstrating that KI dramatically potentiated the photodynamic killing effect. This marks the first report of RBDA mediating effective aPDI against fungal yeast, and suggests that the combination of RBDA and KI can overcome the otherwise limited efficacy of RB-based photosensitization against *C. albicans.*
Wen et al., 2017 [46]	The study investigated the potentiation of RB-aPDI by potassium iodide KI through both in vitro and in vivo experiments. In vitro, microbial suspensions of *Escherichia coli*, *Pseudomonas aeruginosa*, methicillin-resistant *Staphylococcus aureus* (MRSA), and *C. albicans* were divided into several study groups based on treatment conditions: (1) cells treated with RB and KI followed by green light (540 nm), (2) cells treated with RB only plus light, (3) cells treated with KI only plus light, (4) cells treated with RB and KI in the dark, (5) cells added after light exposure of RB and KI, and (6) cells centrifuged after RB incubation before light exposure with KI. In vivo, mice with *P. aeruginosa* skin infections were assigned to four groups: (1) untreated control, (2) RB + KI without light (dark control), (3) RB + light, and (4) RB + KI + light. These groups allowed the researchers to assess the individual and combined antimicrobial effects of RB, KI, and light under varying conditions.	RB demonstrated limited antifungal activity alone against *C. albicans* (only ~1.5 log_10_ reduction at 10 μM RB with 10 J/cm^2^ of 540 nm light), but when combined with 100 mM potassium iodide (KI), it achieved complete eradication (≥6 log_10_ kill) of *C. albicans*. The potentiation effect was only observed when the yeast cells were present during light exposure, indicating the generation of short-lived reactive iodine species such as peroxyiodide, I_2_^•^^−^, and HOO^•^. Notably, *C. albicans* was the only tested organism that showed visible RB fluorescence binding to the cell surface, suggesting partial photosensitizer-cell interaction is crucial for the enhanced killing. The mechanism primarily involved singlet oxygen (^1^O_2_) reacting with iodide to produce iodine species that contribute to microbial inactivation. These findings support the use of RB-KI-mediated aPDI as a highly effective strategy against *C. albicans*, particularly when conventional RB-PDI is insufficient.

aPDT—antimicrobial photodynamic therapy, RB—rose bengal, MB—methylene blue, CUR—curcumin, CFU/mL—colony-forming units per milliliter, PDI—photodynamic inactivation, J/cm^2^—joules per square centimeter, CHX—chlorhexidine, TBO—toluidine blue O, TMPyP—meso-tetra(N-methyl-4-pyridyl)porphine, Zn(II)e6Me—zinc(II) chlorin e6 methyl ester, NaOCl—sodium hypochlorite, EDTA—ethylenediaminetetraacetic acid, EY—eosin Y, PBS—phosphate-buffered saline, LED—light-emitting diode, RBF—riboflavin, APDT—antimicrobial photodynamic therapy, UVA—ultraviolet A, RBDA—rose bengal diacetate, KI—potassium iodide, MRSA—methicillin-resistant Staphylococcus aureus, PDT—photodynamic therapy, P+L+—photosensitizer and light present, P+L−—photosensitizer present, light absent, P−L+—photosensitizer absent, light present, P−L−—photosensitizer and light absent, ATCC—American Type Culture Collection, SC5314—strain designation for *C. albicans*, MYA-2876—ATCC number for *C. albicans* SC5314, I_2_^•^^−^—iodine radical anion, HOO^•^—hydroperoxyl radical, ^1^O_2_—singlet oxygen.

**Table 6:** Light sources physical parameters of studies that fulfilled the eligibility criteria.

Author and Year	Light Source	Wavelength (nm)	Energy Density (Fluence) (J/cm^2^)	Power Output (mW/cm^2^)	Irradiation Time (s)
Soria-Lozano et al., 2015 [35]	Metal Halide Lamp	420–700	37	90	Not stated
Torres-Hurtado et al., 2019 [36]	LED Array	600–650, 490–540	10–60 (single) 3–20 (repetitive)	Not stated	Not stated
Alhenaki et al., 2021 [37]	LED Array	480	37.5	526	180
Diogo et al., 2017 [38]	LED (Red & Green)	627, 557	Not stated	42	90
Freire et al., 2013 [39]	Green LED	532 ± 10	Not stated	237	180
Gavara et al., 2021 [40]	LED (Showtec LED Par 64)	515 ± 10	Up to 200	5.8	Not stated
Silva et al., 2016 [41]	Green LED Array	510–560	10, 20, 30	10	Not stated
Tunçcan et al., 2018 [42]	Green LED Array	518	Not stated	Not stated	Not stated
Hung et al., 2022 [43]	Green LED	532 ± 10	16.2	90	180
Arboleda et al., 2024 [44]	Red LED, Green LED, UVA Lamp	660, 518, 370	5.4, 2.7, 1.8	3	300
Wei et al., 2024 [45]	Green LED	540 ± 15	10–20	20.83	480
Wen et al., 2017 [46]	Green Light	540 ± 15	10–20	100	100

LED: Light-Emitting Diode, UV: Ultraviolet.

**Table 7:** Characteristics of RB used in studies meeting eligibility criteria.

Author and Year	Incubation Time (Minutes)	Concentration/s of RB Used (µg/mL)
Soria-Lozano et al., 2015 [35]	<1, 60, 180	0.31–0.62, 0.62–1.25
Torres-Hurtado et al., 2019 [36]	30	0.51–10.18
Alhenaki et al., 2021 [37]	Not stated	5.09
Diogo et al., 2017 [38]	15	100
Freire et al., 2013 [39]	5	6.36–407.06
Gavara et al., 2021 [40]	Not stated	60
Silva et al., 2016 [41]	15	2000
Tunçcan et al., 2018 [42]	Not stated	1000, 500, 100
Hung et al., 2022 [43]	5	12.72
Arboleda et al., 2024 [44]	15	1000
Wei et al., 2024 [45]	120	407.06
Wen et al., 2017 [46]	Not mentioned	Not mentioned
